# Vision-based Nano Robotic System for High-throughput Non-embedded Cell Cutting

**DOI:** 10.1038/srep22534

**Published:** 2016-03-04

**Authors:** Wanfeng Shang, Haojian Lu, Wenfeng Wan, Toshio Fukuda, Yajing Shen

**Affiliations:** 1 Mechanical Engineering Department, Xi’an University of Science and Technology, Xi’an 710054, China; 2 Department of Mechanical and Biomedical Engineering, City University of Hong Kong, Hong Kong, China; 3 School of Mechatronic Engineering, Beijing Institute of Technology, Beijing 100081, China; 4 City University of Hong Kong Shenzhen Research Institute, Shen Zhen 518057, China

## Abstract

Cell cutting is a significant task in biology study, but the highly productive non-embedded cell cutting is still a big challenge for current techniques. This paper proposes a vision-based nano robotic system and then realizes automatic non-embedded cell cutting with this system. First, the nano robotic system is developed and integrated with a nanoknife inside an environmental scanning electron microscopy (ESEM). Then, the positions of the nanoknife and the single cell are recognized, and the distance between them is calculated dynamically based on image processing. To guarantee the positioning accuracy and the working efficiency, we propose a distance-regulated speed adapting strategy, in which the moving speed is adjusted intelligently based on the distance between the nanoknife and the target cell. The results indicate that the automatic non-embedded cutting is able to be achieved within 1–2 mins with low invasion benefiting from the high precise nanorobot system and the sharp edge of nanoknife. This research paves a way for the high-throughput cell cutting at cell’s natural condition, which is expected to make significant impact on the biology studies, especially for the *in-situ* analysis at cellular and subcellular scale, such as cell interaction investigation, neural signal transduction and low invasive cell surgery.

With the rapid progress of nanobiotechnology, the bio-research at subcellular level has received increasing attentions, since it provides new opportunities for the in-depth understanding of cell’s activities^1^,^2^,^3^. As one of the basic techniques to approach sub-cell analysis, precise cell cutting plays a significant role in many fields. For instance, the cell cutting technique allows us to investigate the biophysical factors in various cell interactions, such as neuron signal transduction^4^, *in-vivo* axon microsurgery^5^ and wound healing^6^. In addition, the cell cutting technique provides a powerful tool for the low invasive cell operation, by which we are able to isolate a single organelle, such as mitochondrion^7^, from a cell, thereby promoting the in-depth understanding of the mitochondria-related bioactivities.

At current stage, the most widely used cell cutting technique is based on cyto microscopy, where a diamond or glass knife is employed to slice the cell embedded in ice or resin^8^. However, in this technique, the sample has to suffer a non-ignorable compression force due to the large edge angle of the diamond or glass knife (usually >25°), which significantly affects the cell’s inner structure and even leads to cell cracking^9^,^10^,^11^. Therefore, although the cyto microscopy is highly effective and productive for the embedded cell cutting in certain environment (−180 °C ~ −60 °C)^12^, it’s not suitable for the cell study under cell’s natural condition.

Atomic force microscope (AFM) is able to separate the connected cells at cell’s natural condition, which has been regarded as a powerful tool for cell cutting at nanoscale^13^. However, it is challenging to directly employ this approach for the individual cell cutting because of the large tip angle of AFM cantilever (usually 45°). Although using the AFM cantilever with a sharp tip could improve the cutting capability, the accurate positioning of the AFM tip is still difficult due to the beam deflection of the AFM cantilever during the cutting process. Moreover, the AFM system doesn’t allow the real-time imaging during the cutting operation because of its point-to-point scanning mechanism, which seriously prevents it from being the highly productive cell cutting system.

Recently, to address the cell cutting task, some new types of nanoknives have been developed by micro-nano fabrication techniques, such as carbon nanotube (CNT) assembly^14^,^15^, microfabrication^5^, and focused ion beam (FIB) etching^16^. Compared with the traditional ones, these novel nanoknives usually have a sharp edge, by which the compression effect on the cell can be reduced greatly. Therefore, they provide new opportunities for the non-embedded cell cutting benefiting from the low physical invasion. Previously, we developed a nanoknife from the AFM cantilever based on FIB etching, which had a sharp tip (5°) and a cutting load ability up to 100 μN^16^. It was successfully used to cut the single cell within an environmental scanning electron microscope (ESEM) and the results showed the nanoknift could cut the cell with less force and less invasion. However, the previous cell cutting process is based on manual manipulation, resulting in a significant amount of time spent in the system operation, including nanoknife positioning, locomotion and cutting operation. In addition, the system has high requirement on the user’s experience and skills, since an improper operation may damage the nanoknife or cell and then cause experiment failure. In short, the low working efficiency and the high operation complexity of current cutting systems have been the main bottlenecks for the high-throughput non-embedded cell cutting and prevented it from being widely acceptable.

Benefiting from the emerging robotic technique, the automatic manipulation at micro-nano scale has become possible^17^,^18^,^19^,^20^,^21^,^22^ and has already been applied to many high-throughput bio studies^23^,^24^,^25^,^26^. Herein, we report a vision-based nano robotic system to address the high-throughput non-embedded single cell cutting task. Firstly, the single cell cutting system is developed by integrating the nanorobot and the nanoknife with an ESEM. Then, the positions of the nanoknife and the single cell are recognized and the distance between them is calculated dynamically based on image processing. To guarantee the system’s position accuracy and working efficiency, we propose a distance-regulated speed adapting strategy, in which the nanoknift’s moving speed is adjusted intelligently according to the distance’ change. Finally, the automatic single cell cutting experiment is demonstrated and the results are discussed.

## Results and Discussion

### Cell cutting system development and calibration

**Figure 1:**
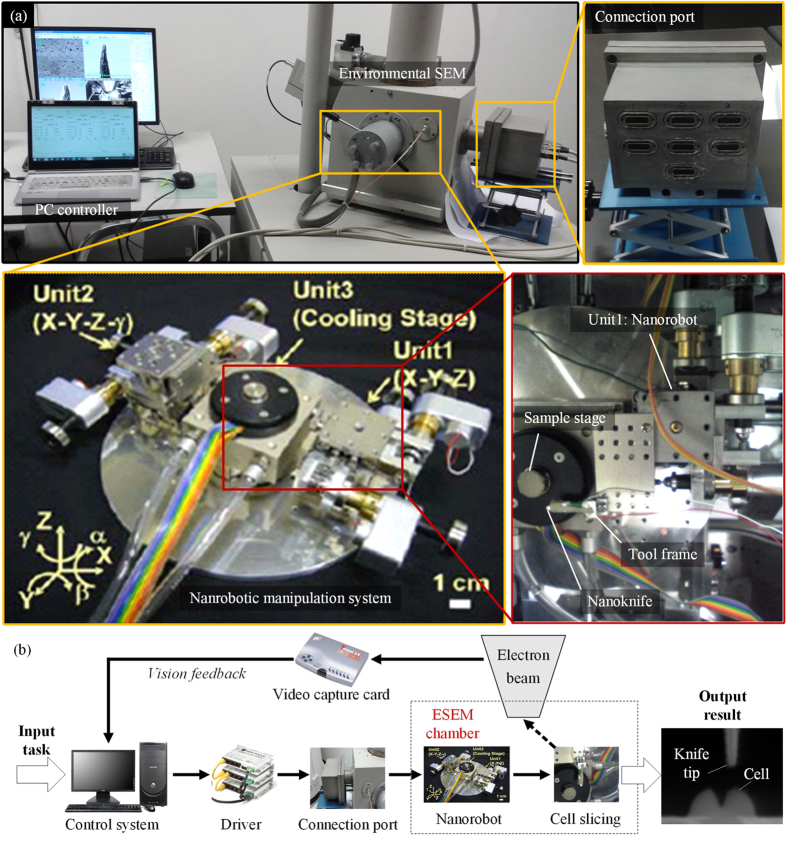
3-DOF nano robotic manipulation system for the automatic cell cutting. (**a**) Overview of the experimental setup. (**b**) Illustration of the automatic system.

As illustrated in Fig. 1, the non-embedded cell cutting system is constructed by integrating the nanorobot, nanoknife, vision-feedback system, PC controller and a connection port with ESEM. The nanoknife is assembled on the nanomanipulation system locating inside the chamber of an ESEM. A specific connection port with 105 conductive pins is designed for the microscopy, through which the nanorobot can be controlled by the PC outside ESEM. An image capture card (Tempest SX VOL-001, Ad-techno Inc.) is used to capture the real-time ESEM images and transmit them to the PC controller. The nanoknife and the cell are recognized and the distance between them is calculated dynamically based on image processing. After that, a proper control signal is sent to the motor driver to control the movement of the nano robotic system through the connection port. As a result, the non-embedded cell can be localized and cut automatically.

**Figure 2:**
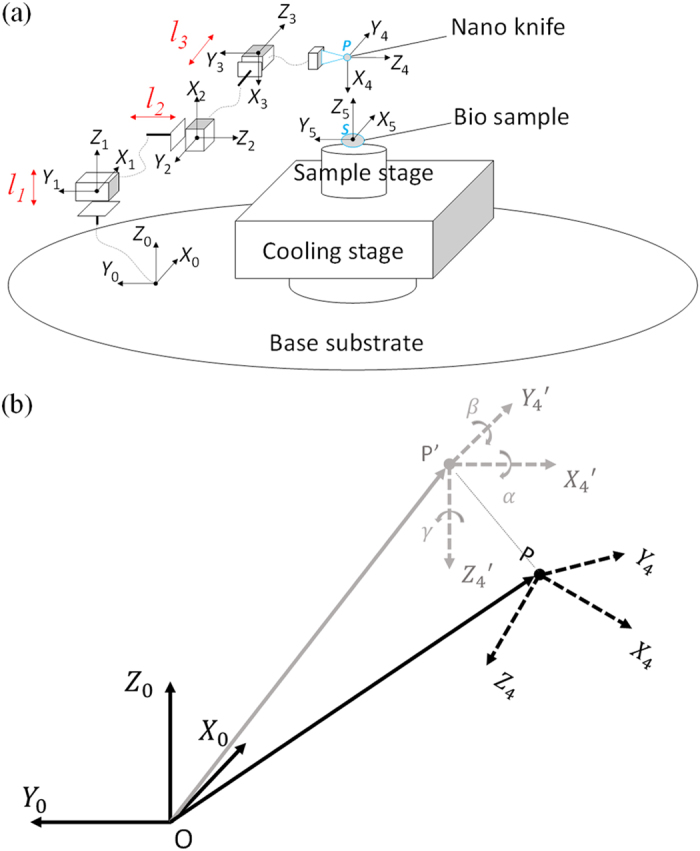
The kinematic model of the nano robotic system. (**a**) Illustration of the nanorobot system. (**b**) Assembly error calibration. *P’* indicates the ideal frame, which is parallel to the sample frame 5. *P* indicates the real frame, which has an assembly alignment error to *P’*.

To describe the locomotion of the nanoknife, the kinematic model of the nanorobotic system is established firstly. As illustrated in Fig. 2a, the base substrate is defined as frame 0. Frame 1, frame 2 and frame 3 are able to translate along *Z*_0_ axis, *Y*_1_ axis and *Y*_2_ axis, respectively. Frame 4 is the tool frame and frame 5 is the sample frame, which are built on the nanoknife and the cell, respectively, in the cell cutting task.

As illustrated in Fig. 2b, there may be some difference between the real tool frame (frame 4) and the ideal frame (frame 4′) due to assembly errors. As a result, the two coordinate frames of the nanoknife (frame 4) and the biosample (frame 5) would also have a rotatory error. In this case, an off-axis error would appear during the operation. For example, during the sample’s movement on the X-Y plane, the Z distance of the sample may also change. To eliminate this off-axis error, the misalignment between the two sets of coordinates must be calibrated in advance.

With point 
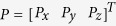
 on the real tool frame (frame 4) and 
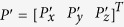
 on the ideal tool frame (frame 4′), the relation between them can be represented by:


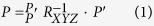


where 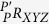 is the rotation matrix that is derived based on Denavit–Hartenberg model^27^:


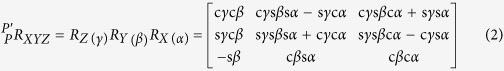


where c and s indicate the symbol of cos and sin, respectively; α, β, and γ are the rotation angle about *x*_4_’, *y*_4_’ and *z*_4_’, respectively. When the nanoknife moves inside ESEM, we can obtain the actual position *P* from the SEM image. Meanwhile, the corresponding ideal position *P’* can be calculated via the following equations:


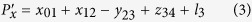



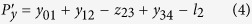



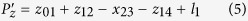


where *x*_(*j−*1)(*j*)_, *y*_(*j−*1)(*j*)_ and *z*_(*j−*1)(*j*)_ (*j = *1, 2, 3, 4) indicate the offset value from frame *j* to frame *j−*1 along axis *x*_*j−*1_*, y*_*j−*1_ *and z*_*j−*1_, respectively. These values are constant once the nano manipulator is constructed. *l*_1_, *l*_2_ and *l*_3_ are the translational distance of the three picomotors, which are variables related to the movement. Suppose we have 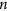 real positions 
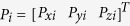
 and *n* corresponding ideal positions 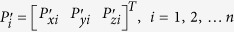. The relation between them can be represented by:


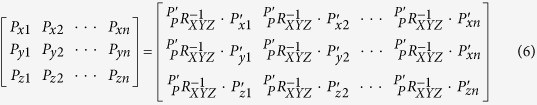


By solving eq. (6), we can find out the values of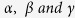, and then the alignment error of the robotic system. Thus, we can adjust the assembly to make 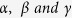 infinitely close to 0 degree. After calibration, the rotation matrix 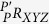 can be simplified to:


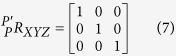


As a result, the product of all four link transformation matrix can be described by homogeneous transformation of each link:


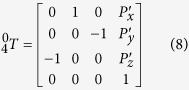


Thus, the tip location of the nanoknife *P* in the base substrate (frame 0) can be described by:


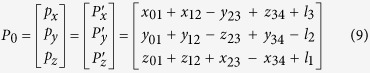


Similarly, the position of the biological sample *S* in the base substrate (frame 0) can be described by:


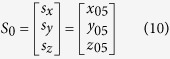


where *x*_05_, *y*_05_, and *z*_05_ are the offset distance from frame 0 to frame 5 along axis *x*_0_, *y*_0_ and *z*_0_, respectively. Thus, the distance *D* between the nanoknife *P*_0_ and the biospecimen *S*_0_ can be described by:


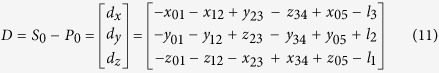


**Figure 3:**
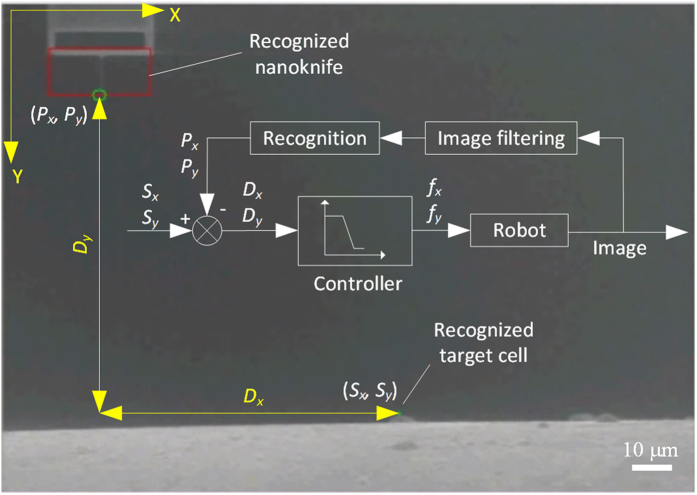
Illustration of the vision-based control system. The distance between the nanoknife’s tip and the cell is taken as the feedback signal for the controller.

It indicates that the movement of the nanoknife on the X-Y plane would not change the Z axis after misalignment calibration. Therefore, we can take *D*_*x*_ and *D*_*y*_ as feedback signal to control the movement on X-Y plane by image processing, as illustrated in Fig. 3.

### Vision-based feedback system

The nanorobot consists of three piezoelectric picomotors, which are able to produce a movement step size less than 30 nm. However, unlike the stepper motor, the picomotor cannot ensure a fixed step size when a fixed width pulse is applied because of the dynamic frictional driving mechanism. This kind of error would accumulate in each step and finally become non-ignorable with time going on, indicating it’s very difficult to locomote the nanoknife precisely based on the open loop system. Therefore, in this paper, we propose a vision-based feedback system to dynamically recognize the positions of nanoknife and the cell, and then take the distance between them as the feedback to automate the cell cutting process.

The positions of the cell and the nanoknife are recognized by two different strategies with regard to their features. For cells, their dimensions and shapes are usually different from each other. In addition, scientists are only interested in some specific cells rather than all of them. Therefore, considering the high variety and uncertainty of the cells, we develop a friendly human-machine interface, in which the target cell can be selected by single clicking (see methods section for details). This approach can not only guarantee the successful rate of the recognition, but also give more flexibility to the users. In contrast, the structure of the nanoknife almost has no change during the locomotion. Therefore, considering nanoknife’s high observability, we propose a hybrid method combining template matching and corner recognition to recognize the position of the nanoknife’s tip, i.e., to locate the tip in the template, and then to dynamically trap the nanoknife by matching the template with the real-time SEM image (see methods section for details).

**Figure 4 f4:**
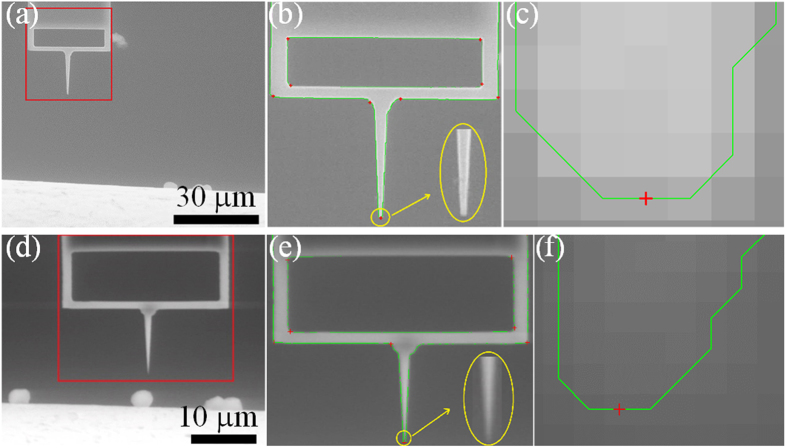
Verification of the nanoknife recognition approach. (**a**–**c**) The first nanoknife at a low magnification. (**d–f**) The second nanoknife at a large magnification.

The errors for the nanoknife recognition mainly come from two parts, i.e., a corner recognition error and a template matching error. For the corner recognition error, the tip of the nanoknife is extracted from the static SEM template based on the CSS (curvature scale space) method. As shown in results of Fig. 4, this recognition error is at a pixel or even a sub-pixel level for different nanoknives regardless of the magnification. The template matching error is also at a pixel level because it’s based on a pointwise match condition^28^. Hence, the maximum recognition error of the nanoknife tip would be no larger than two pixels.

In the experiment, the represented distance of each pixel is calibrated from the known scale bar in the SEM image. Taking Fig. 4 as an example, the recognition accuracy is about 176 nm (equivalent to two pixels) for Fig. 4a and 87 nm (equivalent to two pixels) for Fig. 4d. Note that the feedback vision information highly depends on the resolution of the image, and thereby the positioning accuracy can be improved further if the experiment is implemented at a larger magnification.

### Distance-regulated speed adapting strategy

The main objective of the control system is to move the nanoknife to the target cell. Considering the cell is static and the nanoknife has no any action on the cell during the nanoknife’s movement, there is no need to design a specific movement trajectory for the nanoknife. Therefore, we employ the point to point (PTP) control approach, which is able to guarantee the final positioning accuracy independent of the movement trajectory. In addition, to speed up the movement of the nanoknife to the target cell, we propose a distance-regulated speed adapting strategy for the PTP control, in which different levels of pulse frequency *f*_*P*_ are used to control the nanorobot movement based on the dynamic distance *D*_*i*_ between the nanoknife and the cell:


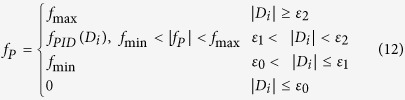


where *i* is a selected axis X or Y (*i* = *x* or *y*); *ε*_0_, *ε*_1_ and *ε*_2_ are user-defined distance thresholds for motor stopping, running at low speed and running at high speed, respectively; *f*_max_ and *f*_min_ are the defined maximum and minimum pulse frequency (Hz or steps/s) driving the motors, which are 250*K* Hz and 10*K* Hz, respectively, with *K* = 1 for “fine speed” and *K* = 8 for “coarse speed” with regard to the operation manual (Picomotor, Newport Inc.). The above control strategy devides the overall movement procedure into four stages: high speed, falling-speed transition, low speed and stopping stages. As a result, the nanoknife can move at a low speed to improve the dynamic performance when it is close to the cell, and keep a high speed to improve the working efficiency when it is far from the cell.

The accuracy of the proposed control system is determined by two factors: the measurement error of the distance *D*_*i*_ and the positioning error of the nanorobot. It has been proved that the error of *D*_*i*_ is less than two-pixel equivalence distance based on the above analysis. For the positioning error of the nanorobot, it is known that the picomotor is an overdamped system. When the control system stops sending speed command and instead sends a “stop” command, the nanoknife driven by picomotor would stop abruptly within a step size. Therefore, the positioning error of the nanorobot would be no more than one step size (30 nm) according to the operation manual. As a result, the maximum error of this control system would be no more than two-pixel equivalence distance plus one step size of the picomotor, e.g., 206 nm and 117 nm for the experiment shown in Fig. 4a[Fig f4]d, respectively. Note that this error can also be reduced by increasing the image magnification.

### Automatic non-embedded cell cutting process demonstration

**Figure 5:**
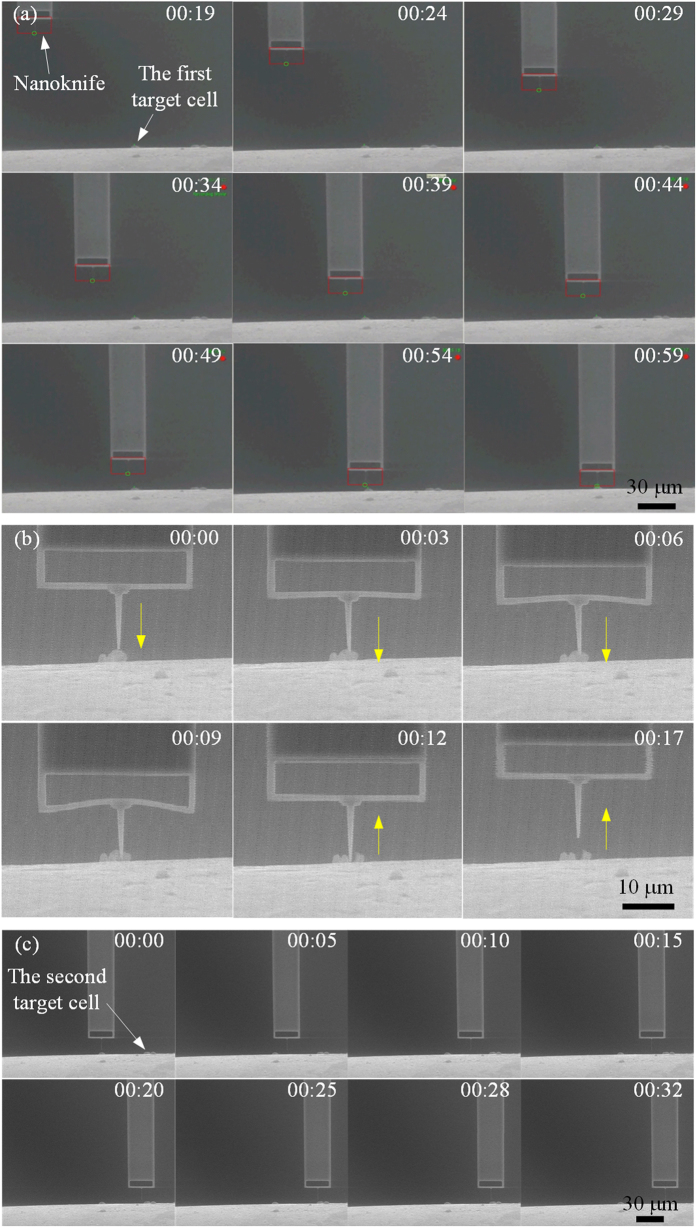
Demonstration of the automatic non-embedded cell cutting process. (**a**) Automatic nanoknife locomotion to the first target cell. (**b**) Cell cutting process. (**c**) Nanoknife locomotion to the second target cell.

In this demonstration, the frequencies *f*_max_ and *f*_min_ are set as 250 Hz and 10 Hz, respectively, and the thresholds are set as follows: *ε*_0_ = 30 nm, *ε*_1_ = 5.0 μm and *ε*_2_ = 30.0 μm. Figure 5a selectively gives nine SEM images (every 5 s) during the locomotion of the nanoknife. At the initial position, the distance between the knife tip and the cell is approximate 87.5 μm and 108.2 μm along X (horizontal) and Y (vertical) axis, respectively. Because these values are larger than *ε*_2_ (30 μm), the two picomotors move at the maximum velocity approximate 4 μm/s (*f*_max_ = 250 Hz) to reduce the operation time. With time going on, the velocity starts to decrease after the distance between the knife tip and the cell is less than *ε*_2_ (30 μm). While the distance becomes less than *ε*_1_ (5 μm), the nanoknife moves at the minimum velocity approximate 0.2 μm/s (*f*_min_ = 10 Hz) to guarantee the dynamic performance. Finally, when the nanoknife tip is much closer to the cell, less than or equal to *ε*_0_ (30 nm), the picomotor driver sends an “abrupt stop” command to stop the nanoknife in one step size and keeps on standby for the next operation.

**Figure 6:**
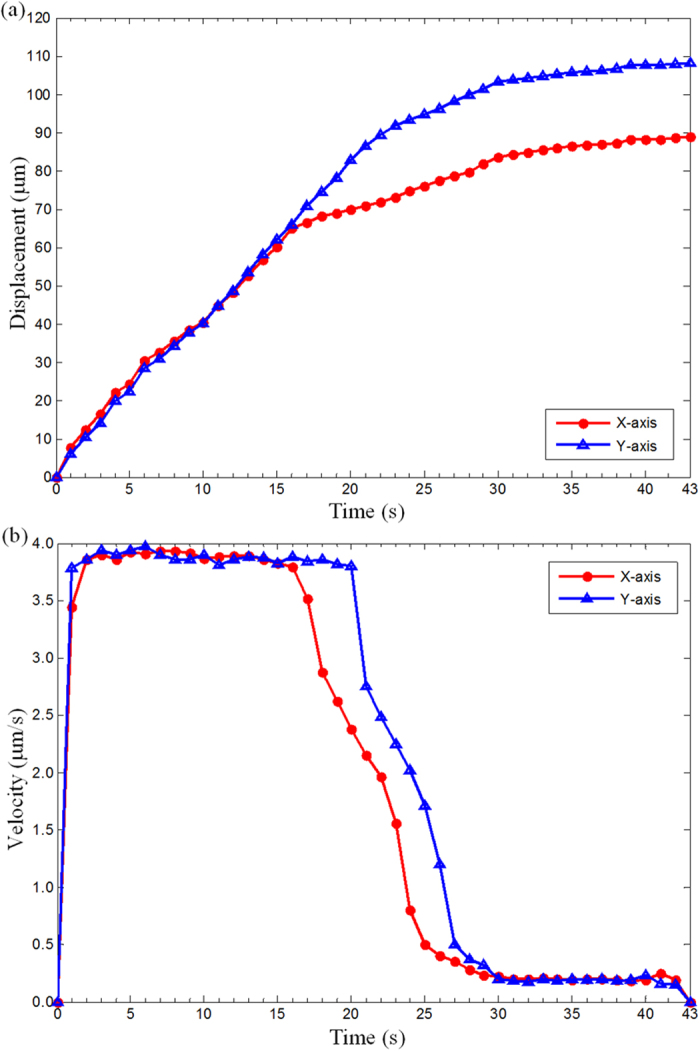
Respond characteristic of vison-based automatic control system against time. (**a**) Displacement vs time. (**b**) Velocity vs time.

The displacement and velocity curve of the nanoknife against time during the locating process is drawn in Fig. 6, which provides us much important information. Firstly, the nanoknife is able to approach the cell along X and Y axis independently, and each curve fits well with the proposed control strategy. Namely, the nanoknife moves faster at the beginning and then slows down when gradually approaching the cell. Secondly, the moving speed doesn’t keep constant although the input pulse frequency (*f*) is the same. We think this is caused by the driven mechanism of the picomotor, in which the fixed width and fixed frequency drive pulse may not result in a fixed displacement because of the dynamic frictional mechanism. Thirdly, as shown in the curves of Fig. 5b, the velocity at low speed (~0.2 μm/s) is more stable than that at high speed (~4 μm/s), which means the movement of the nanoknife has better dynamic performance when it is near the cell. It justifies the necessity and advantages of the distance-regulated speed adapting strategy.

After the nanoknife has moved close to the target cell, user can implement the cell cutting task automatically by single clicking via the operation interface (see methods section for details), after which the nanoknife moves down to cut the cell, and moves back automatically after cutting. As demonstrated by the experiment in Fig. 5b, the velocity and moving down displacement are set to be 0.5 μm/s and 4 μm, respectively. Under this condition, the whole cell cutting process can be completed in 17 s, including moving down and lifting up of the nanoknife. Note that the cutting speed can be increased or decreased by setting different movement speed according to application cases. In addition, benefiting from the sharp edge of the nanoknife, the low physical invasive cell cut can be guaranteed without the requirement of embedded preparation.

Lastly, after the first target cell is cut, the nanoknife then moves to the top of the second target cell automatically by single clicking. As illustrated by the experimental result in Fig. 5c, the nanoknife is able to locate the second cell 65 μm away within about 30 s on the basis of the distance-regulated speed adapting strategy.

Overall, the non-embedded single cell cutting process can be implemented automatically based on the proposed system, including the object recognition, nanoknife locomotion and cell cutting. The results verify that the maximum positioning error of the nanoknife can be limited to two-pixel equivalent distance plus one step size. Secondly, the cutting process for one single cell can be accomplished in less than 2 mins, which is realistic and relatively fast compared with the traditional operation tasks at nano scale, e.g. 5 mins for the scanning of a 5 × 5 μm area by AFM usually and additional operation time for cutting. More importantly, this approach is able to free the users from the complicated nanorobot operation system, and thereby allows them to conduct the single cell cutting experiment easily with high productivity. Note that the running time can be reduced further by selecting “coarse speed” range (*K* = 8) to increase the pulse accounts. In addition, the thresholds are also adaptable based on users’ specific requirements. For example, smaller *ε*_1_ and *ε*_2_ can be adopted if users want to speed up the process further; larger *ε*_1_ and *ε*_2_ can be chosen to improve the dynamic performance of the system to prevent motor from the unexpected runaway.

### Non-embedded cell cutting results

**Figure 7:**
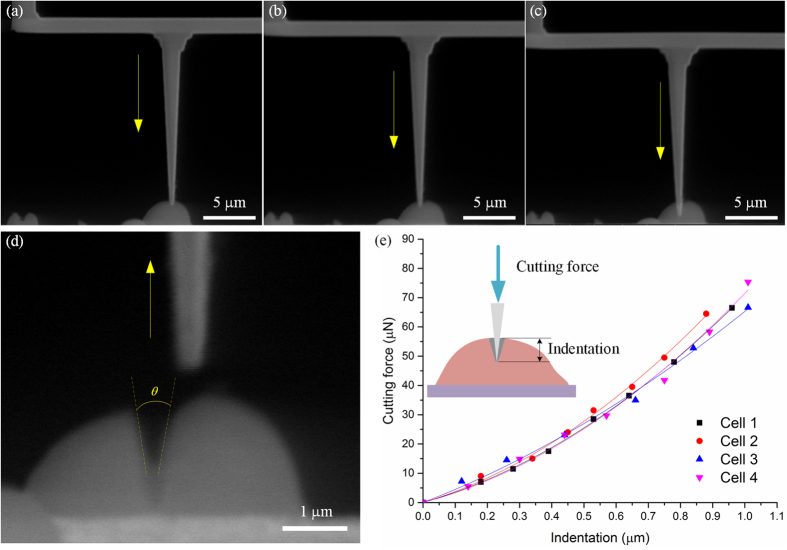
Investigation of the cell cutting process. (**a–c**) Images in the cell cutting. (**d**) Enlargement image of the cell after cutting. (**e**) The typically force-indentation curve before cell separating.

Different from those cells embedded in ice or resin, cells at its natural condition is very soft, and thereby its structure is very sensitive to the applied cutting force. To prevent the cell from rupturing, the cell cutting system should be examined by experiment carefully, especially to evaluate the cutting quality and the cell’s behavior responding to the physical cutting force. Hence, we conduct a series of cell cutting experiments at a constant cutting velocity 0.5μm/s based on the developed system, as shown in Fig. 7. In each experiment, the cutting force exerting on the cell is calculated based on the deflection of the nanoknife’s beam, and the corresponding indentation of the cell is measured from the SEM images. Finally, the cutting force curves against the cell’s indentation for four samples are plotted in Fig. 7e.

It can be seen that the force curves for different cells are very similar, which indicates that the automatic cutting system has high repeatibility. The cutting force increases gradually with the nanoknife impressing, until the cell is separated to two slices while the force reaches near 70 μN. The results show that the cell can be sliced with a small angle θ, approximate 15°, and the angle is even smaller than the edge angle of the traditional knife (usually >25°) for embedded cell cutting. It indicates that the physical invasion to the cell can be controlled at a low level via our system. In addition, compared with other cells, yeast cell is relative difficult to be located and cut due to its small size (~5 μm). Therefore, we believe our cutting system is capable for other types of cells according to the successful yeast cell cutting experiment.

## Discussion

Nowadays, highly productive non-embedded cell cutting has become essentially significant owing to its application in the bioanalysis at cellular and even subcellular level. Herein, in this paper, we propose a unique non-embedded cell cutting system by integrating nanorobot and nanoknife. This system allows us to conduct the cell cutting with high positioning accuracy in a well controllable manner. Moreover, benefiting from the nanoknife, the cell can be cut with low physical invasion.

Subject to the dynamic frictional mechanism, the picomotor doesn’t have precise step repeatability, as a fixed plus cannot ensure a fixed step size. As a result, it is difficult to control the locomotion of the nanorobot precisely by an open loop control system. To address the above issue, we propose a closed loop control system based on vision feedback, through which the nanoknife can be recognized and controlled to move to the target cell precisely. Here, we integrate the template matching and corner recognition approaches to fetch the position of the nanoknife (see methods for details). In this strategy, the position of the nanoknife is calculated from the static templet rather than from the dynamic SEM images. Therefore, the effect of the dynamic disturbance on the recognition accuracy can be ignored during the robot’s moving, including the dynamic image noise, change of the brightness and contrast. As discussed in results section, this hybrid recognition method can ensure the maximum recognition error is less than two-pixel equivalent distance.

Besides object recognition, another significant task for cell cutting is to move the nanoknife to the cell precisely. The proposed PTP control approach has the ability to ensure the final positioning accuracy regardless of the nanoknife’s moving trajectory. In addition, to speed up the operation time, we propose a distance-regulated speed adapting strategy, which is able to control the motor to move at high speed when the distance is large and at low speed when the distance is small. As discussed in the results section, the proposed control strategy is able to guarantee the system accuracy within an error no more than two-pixel equivalent distance plus one step size. Moreover, the control parameters, including the pulse frequency and the thresholds, are adjustable in this method, thereby giving users higher flexibility to optimize the parameters for different application cases. Lastly, the friendly human-machine interface also allows users to operate the complicate cell cutting task more easily than ever before.

The cell cutting results prove that the vision-based automatic control system is able to guarantee both the high-speed locating and the high-accuracy positioning. The total cell cutting process, including object recognition, locomotion and cutting, can be accomplished in less than 2 mins automatically, which is realistic and relative short for nanoscale operation. In comparison, the imaging of a 5 × 5 μm area by AFM usually takes 5 mins, and additional operation time for cutting is required. In addition, the force curves indicate that this method has high cutting reproducibility for different cells. More importantly, this system is able to cut the non-embedded cell with low physical invasion because the cell’s cutting angle is even much smaller than the traditional diamond knife for embedded cell cutting. The above novel features would enable this system to have significant impact in the biology field. For instance, this system can be directly used to cut the neuron of C. elegans with low invasion and then allows us to investigate the neural regeneration dynamically^29^. Moreover, it can also be applied to other analysis tasks of single cell, such as cell manipulation and stiffness characterization. Hence, we believe this automatic system will have significant impact on the biological field, especially on the biology study at cellular and subcellular level.

Nevertheless, this system is still not perfect and need some improvements in the future. First, the developed automatic system only focuses on the 2-D space and the depth information of the object isn’t taken into consideration. In the future, we will develop proper algorithm to estimate the depth of the object and to develop the 3-D control system. Secondly, the parameters in the control strategy are set by the user in advance, which may be difficult for inexperienced users. In the future, we will try to improve the control strategy by employing the adaptive control or other intelligent control methods. Thirdly, we will apply this system to solve one specific problem in biological field, e.g., to study the neutron regeneration by cutting the neutron cell of C. elegans. Lastly, we will extend this system for other applications, such as cell mechanical characterization, cell manipulation and low invasive cell surgery.

## Conclusions

This paper reports a vision-based nano robotic system for the automatic non-embedded cell cutting. This system has high reproducibility and can cut the non-embedded cell with low invasion. In addition, it is able to guarantee both the working efficiency and the positioning accuracy owing to the proposed distance-regulated speed adapting strategy. Moreover, the friendly human-machine interface makes it has high usability even for the inexperienced users. Therefore, it paves the way for the high-throughput non-embedded cell cutting, which is expected to have significant impact on the biology studies, especially on the *in-situ* analysis at cellular and subcellular scale, such as cell interaction investigation, neural signal transduction and low invasive cell surgery. In the future, we will improve this system further by focusing on the 3-D image processing, intelligent control and the multi-functional system development.

## Methods

### Nano robot development

As illustrated in Fig. 1, the nano manipulation system consisted of three units: unit 1 and unit 2 were two sets of nano manipulators, and unit3 was the cooling stage. To achieve the goal of automatic cell cutting, unit 1 and unit 3 were used in this paper. Unit 1 was constructed by three translational picomotors (8301-UHV, Newport Inc.) and a high precision XYZ stage. This unit had three translational degrees of freedom (DOFs) totally with step size less than 30 nm and maximum translation range 12.7 mm. The cooling stage (unit3) was able to adjust the sample temperate from 0 to 40 ^o^C, through which cells are guaranteed to keep their natural condition.

### Nanoknife preparation

The nanoknife was fabricated from a commercial AFM cantilever (OMCL-AC240TM-B2, Olympus Inc.) by FIB etching. The nanoknife had a small tip angle approximate 5°. Also, the buffering beam of the nanoknife can be used to measure the cutting force, and protect the nanoknife from breaking as well. More details about the fabrication process and the advantage of the nanoknife can be found in our previous publication^16^.

### Cell preparation

Yeast cells were cultured on an YPD plate (1% yeast extract, 2% peptone, 2% glucose, 2% agar) for 48 hours at 30 °C in incubator firstly. Then, the cell colonies were diluted in pure water and several drops of solution were put on the tungsten probe substrate using a micropipette. By gradually decreasing the pressure of ESEM chamber, samples can be seen underneath after water evaporates. In this experiment, the acceleration voltage of the electronic beam is set as 15 kV, and the environmental humidity is set as 70%.

### Operation interface

**Figure 8:**
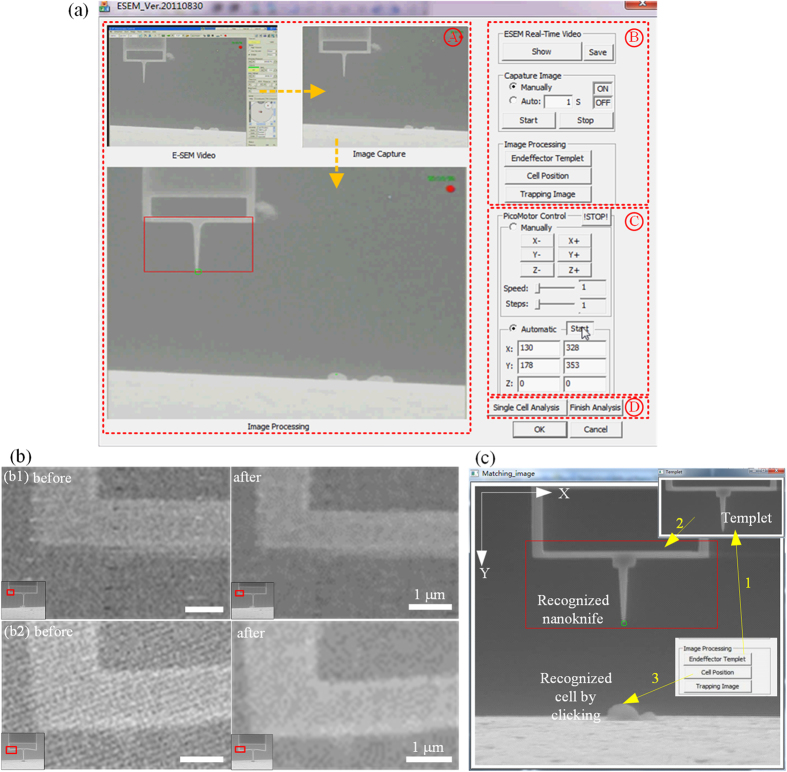
(**a**) Human-machine interface. Region A is for the real-time video monitoring; Region B is for the imaging processing; Region C is for the nanorobot control, and Region D is for the cell analysis, respectively. (**b**) Images comparison between before and after image filtering. (b1,b2) are two examples during the cell cutting experiment: the left images are the original images and the right images are the ones after filtering. The left-bottom window in each picture shows the overall images. (**c**) Illustration of the object recognition. The nanoknife is recognized by template matching. The cell is recognized by single clicking on the human-machine interface. In the developed software, the horizontal and vertical direction is defined as X axis and Y axis, respectively.

A friendly human-machine interface was developed for the automatic cell cutting (Fig. 8a). The left side of the interface was a region showing the real-time experiment video (Region A). The right side was command menus for experiment operation, including image processing (Region B), position control (Region C) and cell cutting (Region D). During the experiment, the real-time video (Region A) was firstly got from the ESEM system by pushing the buttons in Region B. After the position of the nanoknife and the single cell were identified based on image processing, the nanoknife and the single cell were highlighted by two green circles. Then, the nanoknife moved to the target cell automatically driven by the nanorobot (Region C). When the nanoknife reached the target position, the cell cutting was implemented automatically by clicking the “single cell analysis” button (Region D), after which the nanoknife would move down to cut the cell, and move back by itself after the cutting.

### Image noise filtering

We employed a median filter to eliminate the noises in the ESEM images. Theoretically, there are two main types of noise in the ESEM imaging system. The first type is the inherent noises, including the noise due to the statistical nature of electron collision and emission, and noise due to the ESEM detector and signal processing electronics^30^. Generally, most of these noises can be eliminated by the commercial ESEM system itself. The second type of noises is from the external environment, such as mechanical vibration, temperature fluctuation, and electromagnetic field, which is difficult to be eliminated by the ESEM system itself. In the cell cutting system, the main noise is from the movement of the piezo actuator. As shown in the results of Fig. 8b, most of these noises can be eliminated by the designed filter.

### Object recognition and trapping

**Figure 9:**
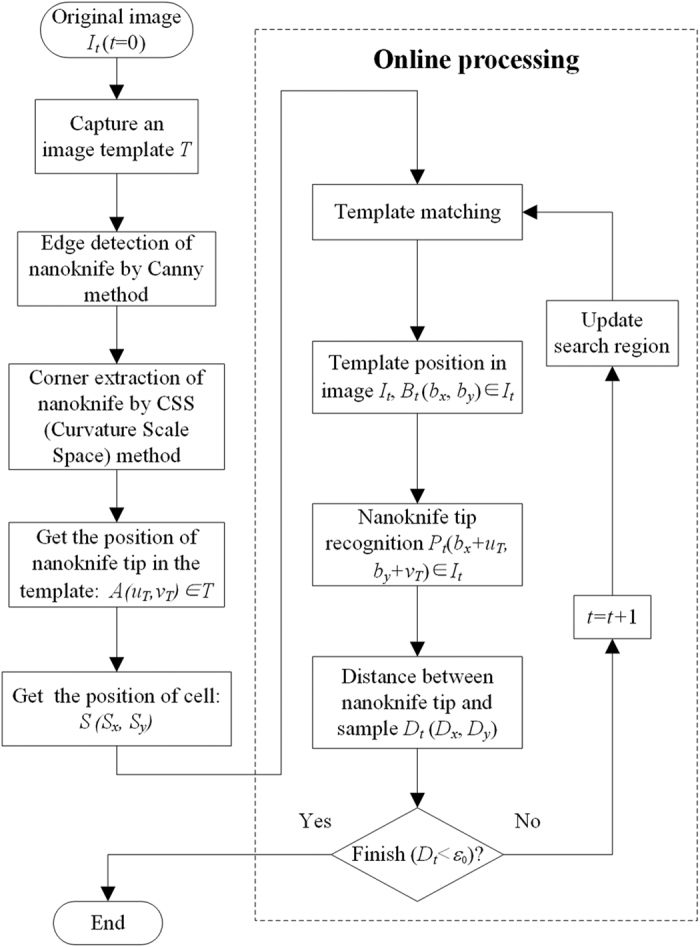
Flow chart for the object recognition and trapping.

The detailed steps of the recognition process were listed in Table 1 and illustrated in Fig. 9. To put it simply, the object recognition consistes of the recognition of the nanoknife and the recognition of the target cell. The target cell was recognized via the human-machine operation interface, through which users can choose the cell and interested region with high flexibility. The nanoknife was recognized based on the hybrid corner detection and template matching. In this approach, the nanoknife tip was firstly extracted from the static template image, through which the corner detection would not be susceptible to the dynamic image noise, change of the brightness and the contract. During the locomotion, the nanoknife was trapped based on template matching, which can not only guarantee the recognition accuracy, but also can reduce computation time.

## Additional Information

**How to cite this article**: Shang, W. *et al.* Vision-based Nano Robotic System for High-throughput Non-embedded Cell Cutting. *Sci. Rep.* **6**, 22534; doi: 10.1038/srep22534 (2016).

## Figures and Tables

**Table 1:** Detailed steps for the object recognition and trapping.

Step 1	Initialize Image 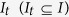 , *t* = 0.
Step 2	Capture an image containing the nanoknife and take it as the template 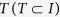 . Then, calculate and initialize the position of template *T*: 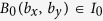 .
Step 3	Extract the nanoknife tip coordinate in the Template *T*: First, extract edge of nanoknife using Canny edge detection algorithm based on maximum local gradient value and judgement rules of adaptive histogram threshold; Second, extract all impossible corners of nanoknife using CSS detection algorithm in variable scale space along the above edge curve. Third, get nanoknife tip corner *A* in the template *T*: 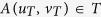
Step 4	Recognize the nanoknife’s tip position *P*_*t*_ at time *t*: First, search in *I*_*t*_ from the last position 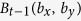 , and match the template *T* to get the new matching position 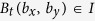 ; Second, calculate the nanoknife’s tip position *P*_*t*_ in *I*_*t*_. Since the nanoknife tip corner *A* in the template is constant, its position *P*_*t*_ in *I*_*t*_ can be represented by *B*_*t*_ + *A*, which is 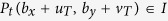 .
Step 5	Calculate the distance between nanoknife and target cell. The sample position *S* (*S*_*x*_, *S*_*y*_) can be obtained via the human-machine interface. So the distance *D*_*t*_(*D*_*x*_, *D*_*y*_) can be represented by: 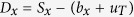 , 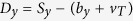 .
Step 6	Reduce search region in the original image, and update 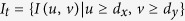 .
Step 7	If *D*_*x*_ < *ε*_0_ and *D*_*y*_ < *ε*_0_, where *ε*_0_ is an arbitrary small positive number, the recognition procedure stops; otherwise set *t* = *t* + 1 and go to step 4.
